# Reactions of Pentafluorohalobenzenes^1^,^2^

**DOI:** 10.6028/jres.063A.010

**Published:** 1959-10-01

**Authors:** Walter J. Pummer, Leo A. Wall

## Abstract

Both pentafluorobromobenzene and pentafluoroiodobenzene are reactive intermediates and can be used to introduce the perfluorophenyl (C_6_F_5_—) group into a variety of new compounds. The chief methods used are the Grignard coupling or addition reaction as well as the Ullmann condensation reaction. Some of the new compounds prepared were pentafluorobenzonitrile, perfluorodiphenyl, pentafluorophenyl-*α*-ethanol, and pentafluorostyrene.

## 1. Introduction

Pentafluorobromobenzene is obtained as one of the products from the synthesis of hexafluorobenzene by the pyrolysis of tribromofluoromethane [1].^3^ The isolation of this material is complicated by the presence of various fluorobromo olefinic and saturated aliphatic compounds boiling in the same range. These undesirable components can be effectively eliminated by subjecting the residue (bp)>110° C) from the initial pyrolysis to another pyrolysis. This procedure converts the contaminants to more hexafluorobenzene and to higher boiling analogs without any apparent destruction of the pentafluorobromobenzene. A relatively pure sample can then be obtained by simple fractionation. Pentafluorobromobenzene and pentafluoroiodobenzene can also be prepared by the bromination or iodination of pentafluorobenzene in 65 percent oleum.^4^

Pentafluorobromobenzene (I) and pentafluoroiodobenzene (III) are useful reactive intermediates Since they contain labile bromine or iodine atoms, these compounds can be used to synthesize numerous new aromatic fluorocarbons containing the perfluorophenyl grouping, C_6_F_5_—. The methods chiefly used are the Grignard coupling or addition reaction and the Ullmann-type condensation. The following scheme will serve to illustrate some of these reactions:

**Figure:**
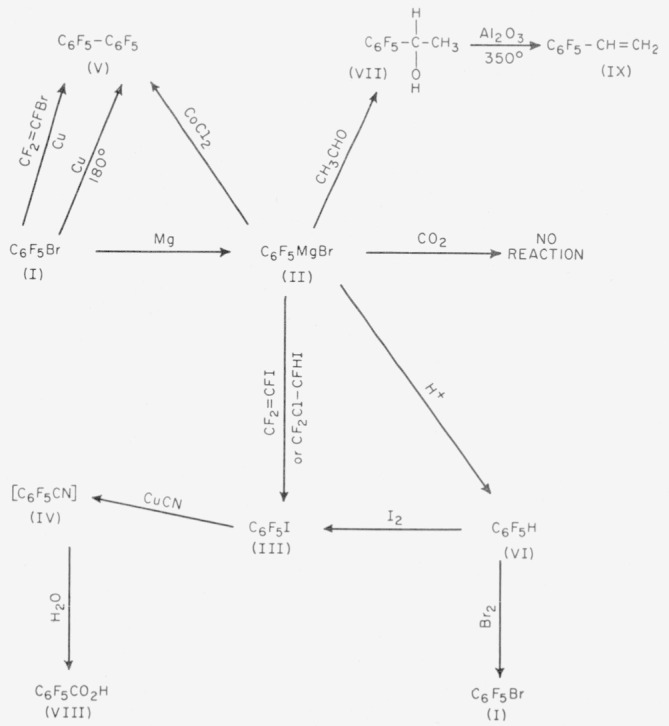


Perfluorodiphenyl (V) was obtained in good yields by the Ullmann condensation of pentafluorobromobenzene (I) in a sealed Pyrex tube at 180° C. The Grignard coupling method with cobalt chloride gave a low yield.

The preparation of perfluorobenzonitrile (IV) directly from pentafluorobromobenzene (I) following the Braun method [2], the use of pyridine and cuprous cyanide, gave unsatisfactory results. The reaction is extremely sensitive to heat, and after a short time at 138° C black insoluble solids appeared and no identifiable products could be obtained. The nitrile (IV) is also obtained from the reaction of (III) and cuprous cyanide. In this case the iodine is even more readily removed than the bromine, and once the reaction was initiated, the products were removed immediately by vacuum distillation. This procedure led to fair yields of pentafluorobenzonitrile (IV), based on its hydrolysis product, pentafluorobenzoic acid (VIII).

Pentafluorobromobenzene (I) readily forms a Grignard reagent, pentafluoroplienylmagnesium bromide (II). Acidification of this reagent yields pentafluorobenzene (VI). The addition of acetaldehyde to (II) forms pentafluorophenyl-*α*-ethanol (VII), which when dehydrated over alumina pellets at 350° C gave pentafluorostyrene (IX). Tarrant [3] employed the coupling of aromatic Grignard reagents and fluorochloro olefins to produce various styrenes halogenated in the side chain. Several attempts were made to utilize this concept for the synthesis of perfluorostyrene via the coupling of (II) and trifluoroiodoethene [4] or its precursor, 1, 1, 2-trifluoro-1-chloro-2-iodoethane. Instead of the desired coupling effect, an exchange reaction occurred, and pentafluoroiodobenzene (III) was obtained.

A similar attempt at preparing perfluorostyrene involved a mixed Ullmann reaction between pentafluorobromobenzene (I) and trifluorovinyl bromide. Only small amounts of perfluorodiphenyl (V) were isolated. No mixed coupling products were observed. Reactions involving the Grignard reagent (II) and tetrafluoroethylene are at present being explored.

Initial attempts to carbonate the reagent (II) by pouring it into an ether-solidified carbon dioxide slurry failed to produce any pentafluorobenzoic acid (VIII) after acidification. Only pentafluorobenzene (VI) was obtained. In this respect the Grignard reagent resembles pentachlorophenylmagnesium chloride in that it also failed to carbonate [5].

## 2. Experimental Procedures^5^

### 2.1. Pentafluorobenzene (VI)

To 1 g (0.041 g-atom) of magnesium turnings in 10 ml of anhydrous ether was added 1 ml of pentafluorobromobenzene (I) and a small crystal of iodine. Local heating was necessary to initiate the reaction. When the reaction was sufficiently in progress the flask was cooled in an ice-water bath and the remainder of compound (I) (total 10 g, 0.04 mole) was added over a half-hour period. The Grignard solution is dark brown in color. When all the magnesium appeared to have been consumed, the solution was allowed to come to room temperature and was stirred for an additional one-half hour. The Grignard solution was then poured on to 50 g of solidified carbon dioxide. After 1 hr, 100 ml of 10 percent hydrochloric acid was added. No pentafluorobenzoic acid (VIII) was observed, but there was obtained 3.5 g (51%) of pentafluorobenzene, bp 85° to 86° C;n_D_^18,9^ = 1.3925; reported bp 88° to 89°; n_D_^18^ = 1.3931 [6].

### 2.2. Pentafluoroiodobsnzene (III)

To 200 g (1.5 moles) of iodine and 1 kg of 65 percent oleum, after stirring for 1 hr at room temperature, was added 255 g of reduced hexafluorobenzene (a mixture containing 45% of hexafluorobenzene, 40% of pentafluorobenzene, and 10% of tetrafluorobenzene) [7]. The mixture was stirred for 4 hr at 55° to 60° C. It was allowed to come to room temperature overnight. The flask was cooled in an ice bath, and gradually 1 liter of ice water was added. It was diluted further with another liter of ice water, then decolorized with aqueous sodium bisulfite, and the products (177 g) separated. After drying (Na_2_SO_4_) and distillation, there was obtained 82 g of unreacted hexafluorobenzene, 36 g of pentafluoroiodobenzene (bp 73° to 75°/35 mm, n_D_^20^ = 1.4990), and 48 g of higher iodinated fluorobenzenes. Analysis—calculated for C_6_F_5_I: C, 24.5; I, 43.1. Found: C, 24.7; I, 42.1.

### 2.3. Perfluorodiphenyl (V) Ullmann Method

Five grams (0.02 mole) of pentafluorobromobenzene (I) and 2.5 g (0.039 g-atom) of activated copper powder [8] were sealed in an evacuated 5-mm Pyrex tube 20-cm long. The tube was placed in a furnace at 180° to 190° C for 48 hr. At the end of this period the temperature was slowly raised to 290° and held at this temperature for 6 hr. It was allowed to cool. Upon cooling, the contents solidified in long white needles. The tube was cooled in liquid nitrogen and opened. There was obtained 2.95 g (87%) of perfluorodiphenyl, mp 63° to 66° C. A recrystallization from methanol, followed by sublimation at 50°/1 mm afforded white plates, mp 67.5° to 68° C. Analysis—calculated for C_1__2_F_10_: C, 43.05. Found: C, 42.9.

### 2.4. Perfluorodiphenyl (V)—Grignard Coupling Method

The Grignard reagent, prepared from 10 g (0.04 mole) of compound (I), 1 g (0.04 g-atom) of magnesium turnings, and 25 ml of anhydrous ether, was filtered into a dropping funnel and added slowly to a refluxing mixture of 0.4 g (0.003 mole) of anhydrous cobalt chloride and 3 ml of ethyl bromide in 3 ml of anhydrous ether. The addition required 45 min. The mixture was then refluxed for 2 hr more, cooled, and acidified with 50 ml of 10 percent hydrochloric acid. The ether layer was separated, dried (Na_2_SO_4_), and distilled. There was obtained 2.5 g (38%) of pentafluorobenzene, bp 84° to 86° C, and 0.5 g of perfluorodiphenyl (V) (15%), mp 67° to 68° C.

### 2.5. Pentafluorophsnyl-*α*-Ethanol Alcohol (VII)

To the Grignard reagent, as prepared from 10 g of compound (I), was added rapidly at 0° C, 6.6 g (0.15 mole) of acetaldehyde. The solution was stirred at 0° C for 1 hr more, and finally decomposed by the addition of 50 ml of 6*N* hydrochloric acid. The ether layer was separated, dried (Na_2_SO_4_), and distilled. There was obtained 7 g (81%) of pentafluorophenyl-*α*-ethanol (VII), bp 80° to 82°/37 mm; $n_{D}^{20}=1.4426$. Analysis-calculated for C_8_H_5_O_5_F: C, 45.3; H, 2.35. Found: C, 44.0; H, 2.30.

### 2.6. Pentafluorostyrene (IX)

Two grams of pentafluorophenyl-*α*-ethanol (VII) was passed through a 10-mm diameter by 20-cm glass tube containing 5 g of alumina pellets [9] at 345° to 350° C with the aid of nitrogen gas. The product (1.4 g) was caught in a solidified carbon dioxide trap and later distilled after drying (Na_2_SO_4_). There was obtained 0.6 g (33%) of pentafluorostyrene (IX), bp 140° to 141°C, $n_{D}^{20}=1.4414$. Mass spectrometer analysis of the product indicated a slight contamination with the starting material (VII).

### 2.7. Attempted Preparation of Perfluorostyrene

(a) The Grignard reagent (II) was prepared from 10 g (0.04 mole) of pentafluorobromobenzene (I), as described previously. To the refluxing Grignard solution was added 9.76 g (0.04 mole) of 1,1,2-trifluoro-1-chloro-2-iodoethane in 5 ml of anhydrous ether. No noticeable heat effect was observed. The solution was refluxed for 3 hr. At the end of this time a black solid appeared on the sides of the flask. The mixture was allowed to stand at room temperature overnight, and finally it was decomposed by addition of 50 ml of 6*N* hydrochloric acid. The ether layer was separated, dried (Na_2_SO_4_), and distilled. There was obtained 5.78 g (50%) of pentafluoroiodobenzene (III), bp 77° to 78°/35 mm, $n_{D}^{20}=1.4990$.

Mass spectrometer analysis of this product showed it to be 98 percent pure. Also, a carbonlike solid, which has not been characterized, was obtained.

(b) The Grignard reagent (II) was prepared from 10 g (0.04 mole) of pentafluorobromobenzene (I) in the usual fashion. To the Grignard solution, maintained at 0° C by means of an ice bath, was added 12.4 g (0.06 mole) of trifluoroiodoethene. The solution was stirred for 1 hr more and then refluxed for 2 hr. Decomposition was effected in the usual fashion. Distillation of the residue, after removal of the ether, afforded 2 g of pentafluorobenzene (VI), 1 g of pentafluoroiodobenzene (III), and 4.5 g of unreacted pentafluorobromobenzene (I).

(c) Two and one-half grams (0.01 mole) of compound (I), 3.71 g (0.06 mole) of activated copper, and 3.22 g (0.02 mole) of trifluorovinyl bromide were sealed in an evacuated 5-mm tube 25-cm long. The tube was placed in a furnace at 100° C, and the temperature was gradually raised to 200° C. This temperature was maintained for 5 hr. No apparent reaction was observed. The temperature was raised slowly to 260° to 270° C, and the tube was kept at this temperature for 15 hr. The copper became straw colored. The tube was cooled in liquid nitrogen and opened. The mixed Ullmann reaction produced only 0.1 g (2%) of perfluorodiphenyl (V). No mixed coupling products were observed.

### 2.8. Perfluorobenzonitrile (IV)

(a) From pentafluorobromobenzene (I), 5 g (0.02 mole) of compound (I), 3.58 g (0.02 mole) of cuprous cyanide, and 1.6 g (0.02 mole) of pyridine were refluxed together for 1 hr. The blackish mixture was cooled, and 50 ml of 6*N* hydrochloric acid was added. There was obtained a carbonlike insoluble solid, along with 30 percent (1.5 g) of recovered pentafluorobromobenzene (I). No pentafluorobenzonitrile (IV) was isolated.

(b) From pentafluoroiodobenzene (III), 5 g (0.017 mole) of compound (III), 1.6 g (0.009 mole) of cuprous cyanide, and 1.34 g (0.017 mole) of pyridine were placed in a small flask and gradually heated. The contents began to darken with increasing temperature. At 150° C the contents were a black viscous liquid. This temperature was maintained for 5 min more. The flask was allowed to cool to 100° C, and the apparatus was rearranged for distillation. The products were removed under reduced pressure obtained by a water aspirator. There was obtained 3.07 g of yellow liquid, bp 185° to 190° C, n_D_^23,8^ = 1.4764. The liquid, on standing, gradually turned brown and probably contained some unreacted starting material (III). To show that the pentafluorobenzonitrile (IV) had indeed formed, half of the above liquid, 1.5 g, was hydrolyzed with 75 percent sulfuric acid at 180° C. Upon pouring on ice, followed by an ether extraction and removal of the solvent, there was obtained 0.2 g (16%, based on reacted material) of pentafluorobenzoic acid (VIII), mp 101° to 103° C (reported mp 104° to 105°) [10]. There was obtained 0.4 g of unreacted starting material.

## Footnotes

3 Figures in the brackets indicate the literature references at the end of this paper.

4 Since the preparation of the article, work similar in part, has been published by E. Nield, R. Stephens, and J. C. Tatlow, J. Chem. Soc. 1959,166.

5 The boiling points given here are not corrected. Chemical analyses were performed by E. Deardorff of the Analytical Section, and the mass spectrometer analyses were obtained by Miss E. Quinn of the Mass Spectrometer Section of NBS.
